# Valley coherent exciton-polaritons in a monolayer semiconductor

**DOI:** 10.1038/s41467-018-07249-z

**Published:** 2018-11-15

**Authors:** S. Dufferwiel, T. P. Lyons, D. D. Solnyshkov, A. A. P. Trichet, A. Catanzaro, F. Withers, G. Malpuech, J. M. Smith, K. S. Novoselov, M. S. Skolnick, D. N. Krizhanovskii, A. I. Tartakovskii

**Affiliations:** 1 Department of Physics and Astronomy, University of Sheffield, Sheffield, S3 7RH UK; 2 Institut Pascal, PHOTON-N2, Université Clermont Auvergne, CNRS, SIGMA Clermont, F-63000 Clermont-Ferrand, France; 3 Department of Materials, University of Oxford, Parks Road, Oxford, OX1 3PH UK; 4 Centre for Graphene Science, CEMPS, University of Exeter, Exeter, EX4 4QF UK; 5 School of Physics and Astronomy, University of Manchester, Manchester, M13 9PL UK

## Abstract

Two-dimensional transition metal dichalcogenides (TMDs) provide a unique possibility to generate and read-out excitonic valley coherence using linearly polarized light, opening the way to valley information transfer between distant systems. However, these excitons have short lifetimes (ps) and efficiently lose their valley coherence via the electron-hole exchange interaction. Here, we show that control of these processes can be gained by embedding a monolayer of WSe_2_ in an optical microcavity, forming part-light-part-matter exciton-polaritons. We demonstrate optical initialization of valley coherent polariton populations, exhibiting luminescence with a linear polarization degree up to 3 times higher than displayed by bare excitons. We utilize an external magnetic field alongside selective exciton-cavity-mode detuning to control the polariton valley pseudospin vector rotation, which reaches 45° at *B* = 8 T. This work provides unique insight into the decoherence mechanisms in TMDs and demonstrates the potential for engineering the valley pseudospin dynamics in monolayer semiconductors embedded in photonic structures.

## Abstract (web summary)

The short exciton life time in atomically thin transition metal dichalcogenides poses limitations to efficient control of the valley pseudospin and coherence. Here, the authors manipulate the exciton coherence in a WSe_2_ monolayer embedded in an optical microcavity in the strong light-matter coupling regime.

## Introduction

**Fig. 1:**
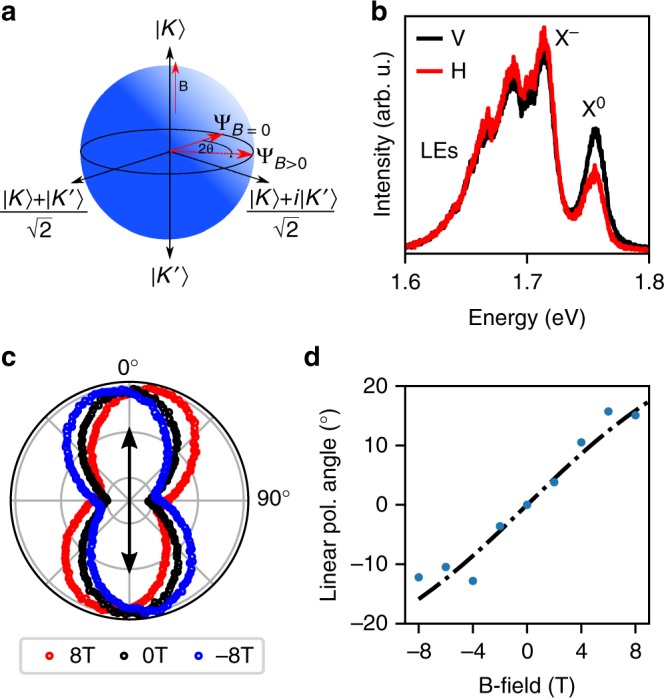
Valley coherence in WSe_2_ excitons. **a** Bloch sphere representation of the valley pseudospin vector. Valley polarized states ($\left| K \right\rangle$ and $\left| {K\prime } \right\rangle$) lie on the poles of the sphere while valley coherence is represented by a Bloch vector oriented on the equator. The application of a perpendicular magnetic field leads to precession of the pseudospin vector around the equator due to the valley Zeeman effect, evolving from a position Ψ_*B*=0_ to Ψ_*B*>0_. **b** Vertically (V) and horizontally (H) polarized spectra under vertically linearly polarized excitation. Retention of injected valley coherence is present for the neutral exciton, X^0^, with a polarization degree of 15%. **c** Rotation of the linear polarization plane around the equator under perpendicular magnetic fields of 8, 0, and −8 T, respectively. The black arrow indicates the injected linear polarization. **d** The linear polarization angle (*θ*) as a function of applied field. Blue circles are data points and dot-dashed black line is a fit corresponding to *θ* = arctan(Ω_B_*T*_2_)/2 where Ω_B_ is the precession frequency given by Ω_B_ = *gμ*_B_*B*/*ħ* and *T*_2_ is the coherence time. The fit corresponds to *T*_2_ = 0.52 ± 0.05 ps

In monolayers of semiconducting transition metal dichalcogenides (TMDs) inversion symmetry breaking, strong spin-orbit coupling and time reversal symmetry lead to the locking of the electronic spin orientation to the specific valley, *K* or *K*′, at the edge of the Brillouin zone^1–3^. This observation has led to renewed interest in valleytronics with proposals to use monolayers of semiconducting TMDs for encoding information in their electronic valley degree of freedom, similar to the approach adopted for spins in spintronics. As with the formalism used for spins, the evolution of the pseudospin can be depicted on a Bloch sphere^1,2,4^, as shown in Fig. 1a, where the poles correspond to states $\left| K \right\rangle$ and $\left| {K\prime } \right\rangle$ with a well defined valley index, and the equatorial plane corresponds to a linear superposition of these states.

It has been expected that owing to the large spin-orbit splittings in TMDs, the valley pseudospin will be robust against intervalley scattering^1,2^. Indeed, optical initialization of the valley states ($\left| K \right\rangle$ and $\left| {K\prime } \right\rangle$) by circularly polarized light has been widely observed for excitons in TMDs such as MoS_2_, WS_2_, and WSe_2_^2,5–8^. In addition, retention of linear polarization has been reported in WSe_2_ and WS_2_, indicating optical initialization of a superposition of valley states $\left| X \right\rangle$ = ${\textstyle{1 \over {\sqrt 2 }}}\left( {\left| K \right\rangle + \left| {K\prime } \right\rangle } \right)$^4,7,9–13^ (see Fig. 1a). However, the lifetime of such exciton valley coherence has been estimated to be a few hundred femtoseconds, limiting the coherent manipulation of the valley pseudospin^4,11–13^. The dephasing has been linked to the random momentum-scattering of excitons on disorder in the presence of the electron-hole exchange interaction^14,15^, in a process known as the Maialle–Silva–Sham (MSS) mechanism^16^. Another factor limiting the ability to manipulate the valley coherence is the short exciton lifetime (1–2 ps)^4,11–13^, which in principle can be overcome by using the valley index of electrons or holes, with the disadvantage of reduced optical control^17,18^.

Recently, an alternative approach enabling new ways to control the valley pseudospin has emerged in experiments on monolayer MoSe_2_ and MoS_2_ embedded in optical microcavities^19–22^, where part-light-part-matter exciton-polaritons are formed due to strong coupling between excitons and the cavity mode. The polaritons exhibit a modified energy spectrum with upper and lower polariton branches split by a few tens of meV and are significantly less sensitive to disorder compared with the excitons. Both effects lead to increased retention of valley polarization in the polariton states^19–22^. In high-finesse tunable microcavities, as in the present work, additional control of the valley pseudospin dynamics can be gained by modifying the exciton-cavity detuning (${\mathrm{\Delta }} = E_{\mathrm{c}} - E_{{\mathrm{X}}^{\mathrm{0}}}$, where *E*_c_ and $E_{{\mathrm{X}}^{\mathrm{0}}}$ are the cavity and exciton energies, respectively), which changes the exciton and photon fractions of the polariton states, thus influencing the polariton radiative and valley depolarization times, as well as modifying exciton energy relaxation^20^.

Here we show that by embedding monolayer WSe_2_ in a tunable microcavity in the strong light-matter coupling regime, we can optically generate valley coherent exciton-polaritons, which are composed of excitons in a coherent linear superposition of valley states which are in a further superposition of exciton and photon states. We demonstrate that valley dephasing can be efficiently circumvented via polariton formation, resulting in a threefold enhancement of linear polarization degree observed for the upper polariton branch (UPB) relative to the bare exciton. On the other hand, the lower polariton branch (LPB) shows a linear polarization degree that is strongly dependent on the exciton-photon detuning. A dynamical model, incorporating cavity-modified exciton relaxation, detuning-dependent polariton lifetimes, as well as disorder scattering in the presence of the excitonic longitudinal-transverse (LT) splitting, reproduces the exciton-cavity detuning dependence of the linear polarization degree. The model and experiments confirm the exciton LT-splitting as the dominant mechanism for exciton dephasing. We then perform coherent manipulation of the polariton valley pseudospin through the application of a magnetic field in the Faraday geometry, leading to a detuning-dependent rotation of the linear polarization plane of polariton emission by angles up to 3 times larger than the bare exciton. This discovery of valley coherent polaritons, which may be non-resonantly optically generated and readily fine-tuned to give desired valley coherence signatures in luminescence, opens the way to unexplored non-linear polariton phenomena utilizing valley coherence in TMDs, such as polariton condensates, the optical spin Hall effect, optical spin switching and polarization bistabilities^23^.

## Results

### Generation and control of exciton valley coherence

The WSe_2_ sample presented here, referred to as sample 1, consists of a single monolayer placed at the surface of a planar dielectric distributed Bragg reflector (DBR). Details and results from a further two samples of identical structure are presented in Supplementary Notes 2, 4, and 5. Characterization of sample 1 was performed under non-resonant excitation at 1.946 eV at 4.2 K and photoluminescence (PL) spectra are shown in Fig. 1b. Features associated with a neutral exciton (X^0^) and charged exciton (X^−^) can be identified along with a large band of localized emitters (LEs) at lower energy. Under vertically linearly polarized excitation (Fig. 1b) the exciton resonance shows retention of linear polarization with a polarization degree of around 15%, indicating the optical initialization of a coherent superposition of valleys. The lack of linear retention for the trion peak has been attributed to the trion fine structure which leads to rapid dephasing of trion coherence^9^. In this sample, a small negative polarization degree of −2% is observed for the trion and is not attributed to valley coherence. Clear retention of valley polarization under circularly polarized excitation is also present (see Supplementary Note 3).

In order to demonstrate control of the valley coherent exciton states we apply a magnetic field in the Faraday geometry^4,12^. This lifts the degeneracy of the exciton valley states $\left| K \right\rangle$ and $\left| {K\prime } \right\rangle$ due to the valley Zeeman effect^24,25^, with the splitting given by *ħ*Ω_B_ = *gμ*_B_*B*. Here, *g* is the exciton g-factor, measured to be −1.7 for this sample (see Supplementary Note 3), Ω_B_ is the Larmor frequency, and *B* the applied magnetic field. After initialization, the linear valley superposition state will evolve with time as $\left| X \right\rangle$ = ${\textstyle{1 \over {\sqrt 2 }}}\left( {\left| K \right\rangle e^{ - {\mathrm{i\Omega }}_{\mathrm{B}}t/2} + \left| {K\prime } \right\rangle e^{{\mathrm{i\Omega }}_{\mathrm{B}}t/2}} \right)$, reaching a new position on the equator of the Bloch sphere with a corresponding rotation of the linear polarization plane. Figure 1c shows the PL intensity as a function of detection angle for applied fields of −8, 0, and +8 T under vertical linear excitation, where clear rotation of the valley coherent pseudospin in PL can be seen. The extracted rotation angle as a function of magnetic field is shown in Fig. 1d. The fit corresponds to *θ* = arctan(Ω_B_*T*_2_)/2 where *T*_2_ is the fitted coherence time, defined as $1{\mathrm{/}}T_2 = 1{\mathrm{/}}2T_1 + 1{\mathrm{/}}T_2^ \ast$ where *T*_1_ and $T_2^ \ast$ are the state lifetime and pure dephasing times, respectively. *T*_2_ is extracted to be 0.52 ± 0.05 ps, in agreement with previous reports^4,11,12^. The dependence of the rotation angle on *g* is consistent with sample 2, in which *g* = −4.1 (Supplementary Note 4).

### Valley coherent exciton-polaritons in WSe_2_ monolayers

**Fig. 2:**
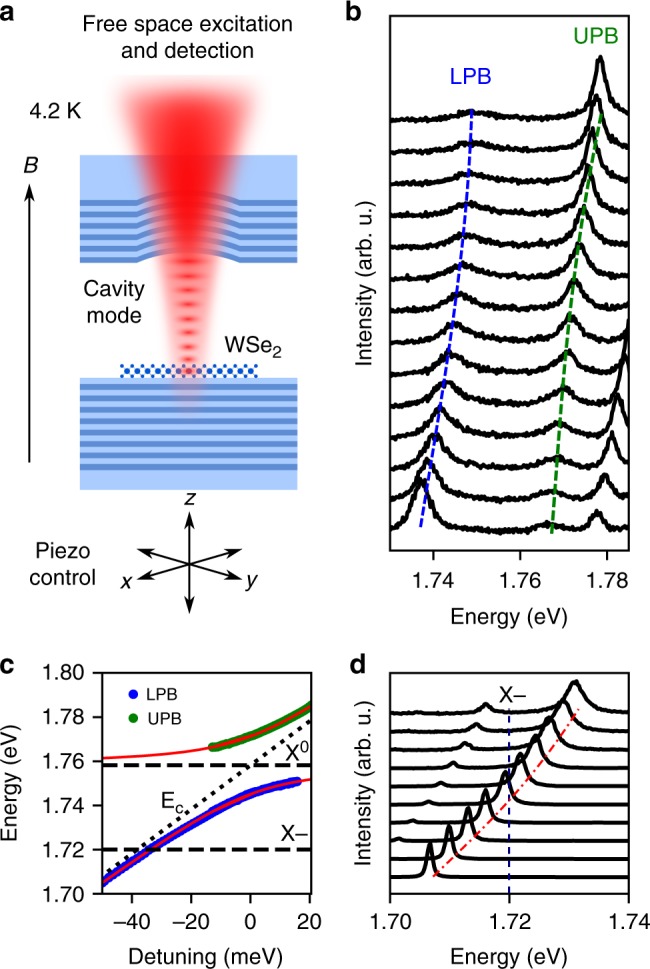
Strong exciton-photon coupling in WSe_2_ monolayers. **a** Schematic of a WSe_2_ monolayer placed in an open hemispherical 0-dimensional microcavity, maintained at 4.2 K in a LHe bath cryostat with superconducting magnet. Piezo nanopositioners allow tuning of cavity length. **b** Photoluminescence spectra measured as the cavity mode is scanned across the exciton resonance, showing the formation of the lower and upper polariton branches, LPB and UPB, respectively. Blue (green) dashed curves guide the approximate peak positions of the LPB (UPB). **c** Extracted polariton peak positions as a function of the exciton-photon detuning. *E*_*c*_ (dotted line) is the energy of the tunable cavity mode. Dashed lines are the energies of the exciton and trion. The green (blue) symbols show spectral positions of the UPB (LPB) peaks. The red lines correspond to a coupled oscillator model fit with a Rabi splitting of 26.2 ± 0.1 meV. **d** Spectra corresponding to tuning the ground state mode through the trion energy, demonstrating weak coupling between the cavity and trion. Blue (red) dashed line indicates the energy of the trion (cavity mode)

The tunable zero-dimensional optical microcavity is formed by introducing a top concave DBR into the optical path using piezo nanopositioners and bringing the two mirrors to a total optical cavity length of around 2.5 μm. A schematic diagram with embedded monolayer is shown in Fig. 2a. The formed microcavity is hemispherical and supports zero-dimensional Laguerre-Gaussian modes. In this work only the coupling with the ground state longitudinal mode is discussed. By changing the mirror separation, the cavity resonance can be scanned across the exciton resonance, allowing observation of a characteristic anti-crossing which signifies the formation of exciton-polariton branches, as shown in Fig. 2b. Fitting the peak positions with a coupled oscillator model yields a Rabi splitting of 26.2 ± 0.1 meV (Fig. 2c). Due to the low intrinsic electron doping present in the sample, weak coupling is observed between the trion and cavity^20,26,27^, as shown in Fig. 2d.

**Fig. 3:**
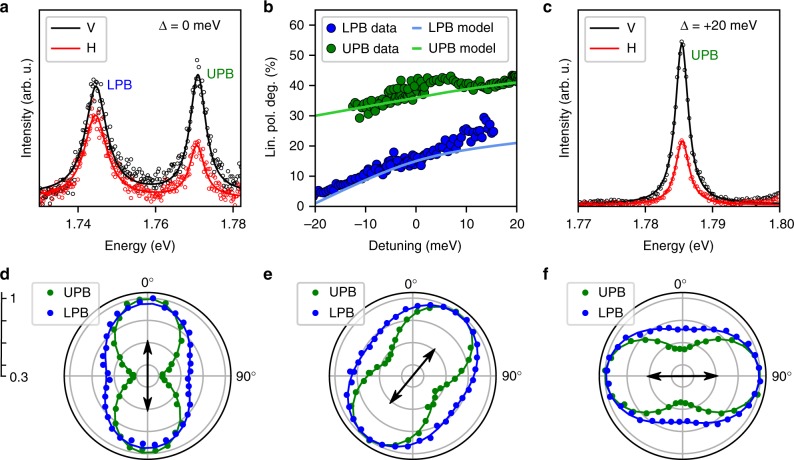
Valley coherence of exciton-polaritons in WSe_2_. **a** Polarization resolved photoluminescence spectra, at zero exciton-cavity detuning (${\mathrm{\Delta }} = E_{\mathrm{c}} - E_{{\mathrm{X}}^{\mathrm{0}}}$) under vertically linearly polarized excitation showing clear retention of injected valley coherence in the polariton branches. Black (red) circles are vertically (horizontally) polarized data points. Solid black and red lines are each a sum of two Lorentzian peaks fitted to the two polariton branches. **b** Linear polarization degree as a function of exciton-photon detuning under vertically linearly polarized excitation for both the LPB (blue circles) and UPB (green circles). The overlaid curves show the simulated polariton polarization degree calculated using the model discussed in the main text. **c** Polarization resolved PL from the UPB at +20 meV positive detuning, demonstrating robust valley coherence and a linewidth of 2.3 meV. **d**–**f** Polariton PL intensity as a function of detection polarization angle for (**d**) vertical, (**e**) diagonal, and (**f**) horizontal linearly polarized laser excitation (indicated by black arrows) at zero exciton-photon detuning. The data for LPB (UPB) are shown with blue (green) symbols. Radial axis is normalized intensity

To probe retention of valley coherence in the polaritonic system we excite at 1.946 eV with vertically linearly polarized light, initially at zero exciton-cavity detuning. Figure 3a shows the polarization resolved PL spectra co- and cross-polarized to excitation. It is clear that both polariton branches show retention of valley coherence, with the UPB displaying a larger linear polarization degree than the LPB. In-situ tunability of the cavity mode energy allows the degree of linear polarization to be probed as a function of exciton-photon detuning, Δ, as shown in Fig. 3b. At large negative detuning the LPB polarization degree is a few percent and increases as the exciton resonance is approached, reaching ≈25% at positive detuning. In contrast, an increase in the UPB polarization degree from 30 to >40% is observed as the detuning is changed from −10 to +20 meV. PL spectra from the UPB at the maximum probed detuning of +20 meV are shown in Fig. 3c, displaying a linear polarization degree 3 times larger than the bare exciton. A large exciton fraction and corresponding low PL emission prevents extraction of the polarization degree in the UPB (LPB) at strong negative (positive) detuning. Measurements on two additional samples show similar behavior of the linear polarization degree as a function of detuning (Supplementary Notes 4 and 5). In order to confirm that the valley coherence is inherited from the non-resonant excitation we rotate the orientation of the linearly polarized pump and record the detection angle dependent PL intensity. The resultant polar plots are shown in Fig. 3d for vertical, diagonal, and horizontal excitation, respectively, where clear retention of the injected valley coherence is demonstrated.

**Fig. 4:**
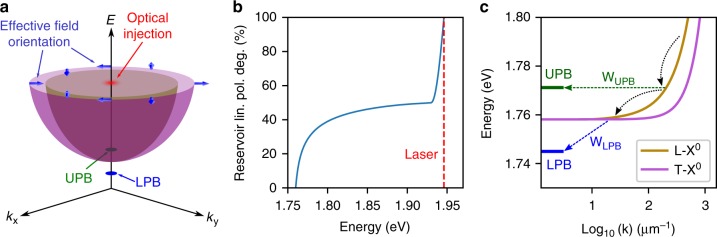
Theoretical model for relaxation of valley coherent excitons and polaritons. **a** Dispersion of polariton and longitudinal-transverse (LT) split exciton states at zero exciton-photon detuning. Carriers are injected at high energy, which form momentum-dark reservoir excitons distributed around the elastic circle. Long range electron-hole exchange interactions lead to a **k**-dependent energy splitting between exciton states with dipole moment parallel (L, represented by the inner parabola) and perpendicular (T, represented by the outer parabola) to the in-plane wavevector. This gives rise to an effective spin-orbit coupling that may be treated as an in-plane magnetic field, which acts on the exciton valley pseudospin and has an orientation which depends upon the exciton momentum. The upper and lower polariton branches (UPB and LPB) exist at lower energy, and are limited to **k** ≈ 0, where the exchange interaction is weak, shielding the polariton states from dephasing. **b** Simulated average linear polarization degree of exciton reservoir states as a function of energy, at zero exciton-photon detuning. The dependence is discussed in the main text. Red dashed line indicates laser energy. **c** Close-up of the dispersion of polariton and LT-split exciton states, at zero exciton-photon detuning. Dashed and dotted lines indicate relaxation pathways of excitons, with *W*_UPB_ and *W*_LPB_ describing the relaxation rates from the reservoir into the polariton branches

Alongside the experimental data, Fig. 3b shows simulated polariton polarization degrees (mathematical details of the simulation are given in Supplementary Note 1). The simulation is based on a model that takes into account coupling between $\left| K \right\rangle$ and $\left| {K\prime } \right\rangle$ valley excitons via the long range electron-hole exchange interaction, which causes the exciton dispersion to split into components with dipole moment parallel (L) and perpendicular (T) to the in-plane wavevector. This LT-splitting is linearly proportional to the **k**-vector outside the light cone, and gives rise to an effective spin-orbit coupling which may be treated as an in-plane magnetic field acting on the exciton valley pseudospin, with field strength and orientation determined by the **k**-vector (Fig. 4a)^14–16,28,29^. Scattering of the excitons due to disorder creates a randomly varying field which leads to random valley pseudospin precession and associated dephasing^4,11–13^. In the model, linearly polarized excitation optically generates valley coherent carriers which scatter to form high in-plane **k**-vector excitons at the pump energy of 1.946 eV. Here, these excitons are considered as occupying the highest energy states of a momentum-dark reservoir, where they have a uniform pseudospin orientation and an even distribution over the elastic circle. At these energies, the spin-orbit coupling is large, which ensures that the pseudospin precession about the **k**-dependent effective magnetic field is much faster than the momentum scattering induced by disorder. Consequently, the states with pump-induced pseudospin parallel to the effective field remain 100 % polarized, while states perpendicular rapidly drop to 0 % mean polarization. Therefore, when integrating over the elastic circle, the reservoir polarization degree averages to 50% very soon after optical injection (Fig. 4b), despite a slow depolarization time limited by inefficient disorder-assisted scattering at such high energy. At lower energies, the reservoir polarization degree decreases because of two reasons. Firstly, exciton states are no longer directly populated by the pump, but also by multi-phonon scattering. Secondly, disorder scattering gets faster and becomes comparable to the pseudospin precession time. These two processes combine to provide an increasingly efficient depolarization effect towards the bottom of the reservoir (Fig. 4b). At some point during their energy relaxation, excitons will scatter from the reservoir into the UPB or LPB, as displayed in Fig. 4c. While some dephasing will occur in the polariton states, it is inefficient due to their insensitivity to disorder, reduced exciton fraction, and low **k**-vector enforced by the 0-D cavity modes^20^. Upon radiative decay, allowed by the photonic fraction of the polariton states, the degree of linear polarization quantifies the level of preservation of valley coherence during the relaxation process.

Under this interpretation, the detuning dependences of the polarization degrees in Fig. 3b become clear. Considering first the LPB, which is populated by excitons which have accumulated at the bottom of the reservoir, a low polarization degree is expected at negative detunings as the large energy separation between the LPB and reservoir, along with a low excitonic fraction, suppresses the relaxation rate *W*_*LPB*_. As detuning increases, and the exciton fraction with it, *W*_*LPB*_ correspondingly increases, allowing reservoir excitons to populate the LPB over a shorter timescale, reducing their exposure to spin-orbit induced dephasing. The UPB, in contrast to the LPB, is always degenerate with some portion of the parabolic exciton dispersion (Fig. 4a), allowing it to be populated by direct disorder-associated scattering of reservoir excitons. This relaxation pathway is fundamentally different to that of the LPB, and explains the overall enhanced retention of valley coherence seen in both the simulated and experimentally observed UPB polarization degrees in Fig. 3b. Even at strong negative detuning when the UPB is highly excitonic, it exhibits robust valley coherence double that of the bare exciton, thanks to the large 26 meV Rabi splitting ensuring that the UPB always remains at least a few meV above the bottom of the exciton reservoir. As detuning increases, the UPB moves to higher energy, so its polarization degree reflects the changing pseudospin relaxation dynamics in progressively higher energy reservoir states. Initially moving from negative to positive detuning, the polarization degree increases as the UPB becomes resonant with higher energy reservoir excitons with stronger valley coherence. At larger positive detuning, the UPB polarization degree saturates as it approaches 50%, which corresponds to the momentum-averaged reservoir polarization degree at high energies.

### External manipulation of exciton-polariton valley coherence

**Fig. 5:**
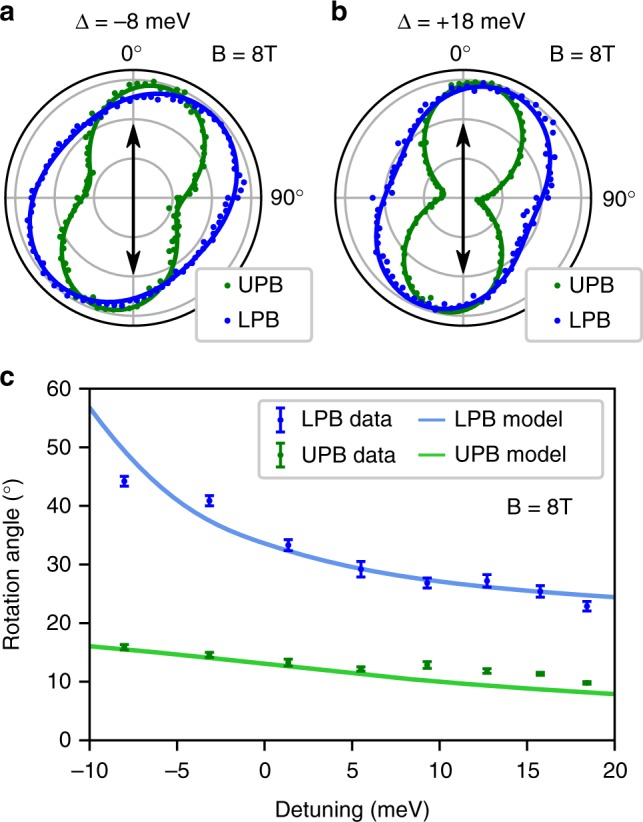
Coherent control of the exciton-polariton valley pseudospin vector under a magnetic field. **a**, **b** Polariton intensity as a function of the linear polarization angle in detection under a magnetic field of *B* = 8 T in the Faraday geometry, for detunings of **a** Δ = −8 meV and **b** Δ = +18 meV. The black arrows indicate the injected linear polarization. **c** The rotation angle of the linear polarization plane of polariton PL as a function of the exciton-photon detuning at *B* = 8 T, under vertical linear excitation. The data for LPB (UPB) are shown in blue (green). Errorbars correspond to the error in fitting the detection angle dependent intensity data at each detuning value. Overlaid curves are simulated rotation angles, calculated using the model described in the main text

To demonstrate control of the valley coherent polariton population we apply a magnetic field in the Faraday geometry as discussed previously for the bare exciton. Figure 5a, b show the polariton PL as a function of detection angle under vertical excitation and at exciton-photon detunings of −8 and +18 meV, respectively, for an applied magnetic field of *B* = 8 T. It is clear from the plots that the induced rotation of the LPB is much larger than that of the UPB, the latter being comparable to the bare exciton rotation. In order to probe the effect of exciton-photon detuning we plot the rotation angle of the LPB and UPB at a fixed field of *B* = 8 T and sweep the detuning from −10 to +20 meV. The resultant rotation angles are plotted in Fig. 5c where a clear increase in the rotation angle is present for increasing negative detuning. Significantly, the LPB rotation approaches 50° at −10 meV, a factor of almost 3 times larger than the bare exciton. At detunings below −10 meV, the small orthogonal polarization of the bare trion masks any coherent rotation of the LPB, as discussed in Supplementary Note 6.

Alongside the experimental data, Fig. 5c also shows simulated polarization rotation angles, calculated from the model discussed above regarding the linear polarization degree, with a modification to include external magnetic field induced pseudospin precession (details can be found in Supplementary Note 1). In this case, a larger angle of rotation in the final state photon reflects a slower overall relaxation pathway taken by the initial state exciton. The UPB extracts high **k**-vector excitons from the reservoir before significant pseudospin precession has occurred, while the excitons which accumulate at the bottom of the reservoir eventually populate the LPB at a rate determined by the detuning. The majority of pseudospin vector rotation occurs in the reservoir, due to the reduced exciton fraction of polaritons, which leads to smaller Zeeman splitting and a lower precession frequency than pure excitons.

## Discussion

In conclusion, we report the generation and control of valley coherent exciton-polaritons in a WSe_2_ monolayer embedded in a microcavity. Our phenomenological model, together with experimental measurements, confirms that the exciton LT-splitting, arising from the long-range Coulomb exchange interaction, is the dominant mechanism of exciton dephasing. By utilizing the strong light-matter coupling regime, we efficiently transfer excitons exhibiting strong valley coherence from high to very low momenta, where they populate polariton states which are exceptionally resistant to valley dephasing. Simultaneously, we probe the interplay between valley dynamics and energy-momentum relaxation in otherwise dark exciton reservoir states over a wide energy range, revealing that the fundamentally different relaxation pathways into the UPB and LPB result in their distinct polarization responses to the externally controlled exciton-cavity detuning. Furthermore, we demonstrate detuning-dependent coherent manipulation of the polariton pseudospin vector. While in this case magnetic fields were used, optical pulses as used in^13^ would allow high speed rotation of the vector to an angle which may be accurately externally controlled via the exciton-cavity detuning.

This observation of robust valley coherence in the strong coupling regime opens tantalizing possibilities to control the valley pseudospin by exploiting the various advantages polaritons have to offer beyond excitons. For instance, polaritons are highly coherent states which have extremely fast propagation speeds and a low effective mass thanks to their photon fraction, yet they also inherit strong interactions and non-linearity from their exciton component. They have an extended spatial wavefunction compared to excitons and hence feel a much reduced effect of the short range disorder potentials currently found in TMD monolayers. Furthermore, polaritons are far more amenable to external control than excitons, as their properties depend so heavily on the specific design of the photonic structure in which they are embedded. This is exemplified by our findings, where UPB states with strong valley coherence are arbitrarily generated at any chosen energy over a 30 meV range, while retaining PL linewidths less than half the bare exciton linewidth, and remaining spectrally isolated thanks to the modified photonic density of states enforced by the cavity. Moreover, the cavity-enhanced polariton lifetimes (5–10 ps, depending on the detuning) are several times longer than the bare exciton (≈1 ps), allowing improved coherent pseudospin manipulation using Stark pulses. We anticipate that TMD-based polaritonic circuit elements may be used for valleytronic devices exploiting controlled polariton pseudospin dynamics, as shown in this work. Such valley polaritonic systems allow for the possibility of extended spatial propagation in two-dimensional microcavities and waveguides, as well as the potential for response to electric fields (particularly for trion-polaritons) with low sensitivity to disorder and, importantly, a strong non-linearity at high polariton densities.

## Methods

### Sample preparation

Monolayer sheets of WSe_2_ were obtained through mechanical exfoliation of bulk crystals. A monolayer of WSe_2_ was transferred onto the DBR surface using standard mechanical exfoliation and standard transfer techniques. Bulk crystals were acquired from HQGraphene.

### Experiments on several WSe_2_ monolayer samples

The results presented here were reproduced by 2 additional samples of the same design. Results from samples 2 and 3 can be found in Supplementary Notes 4 and 5, respectively. The theoretical model was applied to all samples, resulting in accurate fitting of the linear polarization degree as a function of detuning in each case, with only minor adjustments to model parameters. A list of parameters may be found in Supplementary Note 2.

### Optical measurements

Optical measurements were performed with samples held in a helium bath cryostat system at a temperature of 4.2 K. Top and bottom DBRs were attached to XYZ nanopositioners with additional goniometer stages allowing tilt control of the bottom DBR. Optical excitation of the bare monolayer was possible by removing the top DBR from the optical path. All μ-PL experiments were performed with a continuous-wave (cw) excitation using a 638 nm laser diode, focused onto the sample with an achromatic lens. Polarization resolved measurements were performed using a combination of linear polarizer and a quarter waveplate in the excitation path, and quarter waveplate, half-wave plate and linear polarizer in the collection path, allowing linearly and circularly polarized excitation and detection. PL was collected by focusing onto a single mode fiber which was guided into a 0.75 m spectrometer and a high sensitivity charge-coupled device.

### Microcavity

The tunable microcavity with embedded TMD monolayer is formed using an external concave mirror to produce a zero-dimensional tunable cavity^30,31^. The formed cavity schematic is shown in Fig. 2a with the monolayer placed at an electric-field antinode, and nanopositioners are used to control the cavity spectral resonance energy. The nominal radius of curvature of the concave mirror is 20 μm leading to a beam waist on the planar mirror of around 1 μm^30^.

## Electronic supplementary material

Supplementary Information

## Data Availability

All data relevant to this work is available on request to the corresponding author.
