# Supplementary material for: Valley coherent exciton-polaritons in a monolayer semiconductor
Source: Nat Commun. 2018 Nov 15;9:4797. doi: 10.1038/s41467-018-07249-z (PMC6237922; doi:10.1038/s41467-018-07249-z)
Supplement: Supplementary file 1 — Supplementary Information [file 41467_2018_7249_MOESM1_ESM.pdf]

Supplementary Information: Valley coherent exciton-polaritons in a monolayer semiconductor

S. Dufferwiel,[1,*] T. P. Lyons,[1,†] D. D. Solnyshkov,[2] A. A. P. Trichet,[3] A. Catanzaro,[1] F. Withers,[4] G. Malpuech,[2] J. M. Smith,[3] K. S. Novoselov,[5] M. S. Skolnick,[1] D. N. Krizhanovskii,[1] and A. I. Tartakovskii[1,‡]
[1]*Department of Physics and Astronomy, University of Sheffield, Sheffield S3 7RH, UK*
[2]*Institut Pascal, PHOTON-N2, Université Clermont Auvergne, CNRS, SIGMA Clermont, F-63000 Clermont-Ferrand, France*
[3]*Department of Materials, University of Oxford, Parks Road, Oxford OX1 3PH, UK*
[4]*Centre for Graphene Science, CEMPS, University of Exeter, Exeter, EX4 4QF, UK*
[5]*School of Physics and Astronomy, University of Manchester, Manchester M13 9PL, UK*
(Dated: November 1, 2018)

SUPPLEMENTARY NOTE 1: A MODEL OF SPIN DYNAMICS IN STRONGLY-COUPLED TMDS

Spin relaxation model

To describe the spin dynamics in presence of various effective and real fields, we represent the exciton reservoir as a set of states corresponding to different energies E and different propagation directions α. Each state is described by the population $n(E,\alpha)$ and pseudospin $\mathbf{S}(E,\alpha)$. We assume that while the pseudospin distribution may indeed be different for different angles α, populations are the same: $n = n(E)$. Two extra states describe the lower and upper polaritons, for which we also define the populations $n_{L/U}$ and the pseudospins $\mathbf{S}_{L/U}$. The pseudospin is normalized in such a way that its ratio to the number of particles gives a corresponding polarization degree: $\rho_i = S_i/n$.

The excitons in the reservoir are affected by the spin-orbit coupling, which results in a effective magnetic field determined by their propagation direction and velocity:

$$\mathbf{\Omega}_{SOC}(\mathbf{k}) = \beta k(\cos 2\alpha, \sin 2\alpha, 0)^T \tag{1}$$

where α is the propagation direction and β is the spin-orbit coupling constant.

The rate equations for the populations of the reservoir states are written as:

$$\begin{aligned}\frac{d}{dt}n\left(E\right) = P\left(E\right) - \Gamma n\left(E\right) + \textstyle\sum_{E'} W\left(E'-E\right)n\left(E'\right)\\ -\left(W\left(E-E'\right)+W_L\left(E\right)+W_U\left(E\right)\right)n\left(E\right)\end{aligned} \tag{2}$$

where $P(E)$ describes pumping, Γ is the non-radiative decay and the phonon-assisted scattering rates are given by:

$$W\left(E'-E>0\right) = W_{ph}\left(1+\exp\left(\frac{E-E'}{k_BT}\right)\right) \tag{3}$$

$$W\left(E'-E<0\right) = W_{ph}\exp\left(\frac{E'-E}{k_BT}\right) \tag{4}$$

and the scattering rates towards the polariton branches are given by

$$W_U\left(E\right) = W_{U0}\delta\left(E-E_U\right) \tag{5}$$

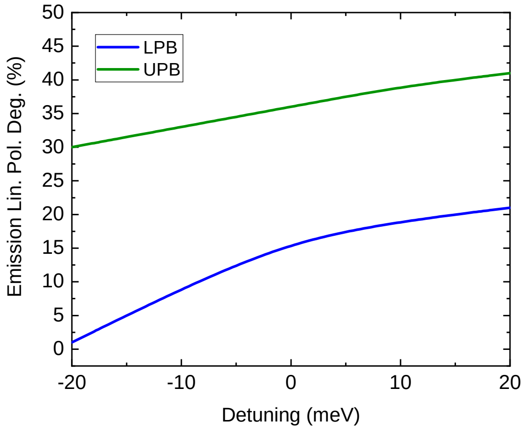

Supplementary Figure 1. Linear polarization degree of the polariton modes (blue - LPB, green - UPB) as a function of the exciton-photon detuning.

and

$$W_L\left(E\right) = W_{L0}x_L\exp\left(\frac{-(E-E_L)^2}{2\sigma^2}\right) \tag{6}$$

where E_L and E_U are the energies of the lower and upper polariton modes, x_L is the excitonic fraction of the lower polariton. All these parameters depend on the detuning Δ. Finally, σ is the inhomogeneous broadening of the exciton line, which allows the excitons to scatter quite efficiently to the lower polariton mode, in spite of the fact that the latter is quite far. However, this scattering rate strongly decreases at negative detunings. The upper polariton is described as being resonantly populated from the reservoir mode which has the same energy, as ensured by the Dirac's delta function.

The rate equations for the pseudospin take into account not only the scattering between different energies, but also the rotation of the pseudospin around effective

magnetic fields (spin-orbit coupling Ω_{SOC} and Zeeman field Ω_Z) and the scattering between the different propagation directions caused by disorder.

$$\begin{array}{c}\frac{d}{dt}\mathbf{S}\left(E,\alpha\right)=\mathbf{P}\left(E\right)+\mathbf{\Omega}\left(E,\alpha\right)\times\mathbf{S}\left(E,\alpha\right)\\ +W_d\left(E\right)\left(\left\langle\mathbf{S}\left(E\right)\right\rangle-\mathbf{S}\left(E,\alpha\right)\right)\\ +\sum_{E'}W\left(E'-E\right)\mathbf{S}\left(E',\alpha\right)-\Gamma\mathbf{S}\left(E,\alpha\right)\\ -\left(W\left(E-E'\right)+W_L\left(E\right)+W_U\left(E\right)\right)\mathbf{S}\left(E,\alpha\right)\end{array} \quad (7)$$

Here, $\mathbf{\Omega}$ includes both the spin-orbit coupling $\mathbf{\Omega}(\mathbf{k})$ defined above and the Zeeman splitting Ω_Z if the latter is applied. The disorder-induced scattering W_d couples all states with the same energy E. Its contribution tends to fill a state with a given direction α by particles having the spin equal to its average value $\langle\mathbf{S}(E)\rangle_\alpha$. The terms with W are identical to those of the equations for the populations and keep the required normalization of the pseudospin: they do not affect the polarization degree and they cannot lead to the rotation of a pseudospin.

The rate equations for the populations of the polariton states read:

$$\frac{d}{dt}n_{L/U}=\sum_E W_{L/U}\left(E\right)n\left(E\right)-\frac{n_{L/U}}{\tau_{L/U}} \quad (8)$$

and for pseudospins:

$$\frac{d}{dt}\mathbf{S}_{L/U}=\sum_E W_{L/U}\left(E\right)\left\langle\mathbf{S}\left(E\right)\right\rangle-\frac{\mathbf{S}_{L/U}}{\tau_{L/U}}+\mathbf{\Omega}_{L/U}\times\mathbf{S}_{L/U} \quad (9)$$

In our simulations, we have used the following parameters: 1) the spin-orbit coupling constant $\beta=30$ μeV$\cdot\mu m$, 2) the disorder-induced scattering $W_d=2.8\times10^{13}$ s^{-1} (this was one of the fitting parameters), 3) the value of the Zeeman splitting from the experiment, 4) the energy relaxation rate $W_{ph}=7\times10^{14}$ s^{-1} (another fitting parameter), 5) the scattering rate towards the LPB W_{L0} was taken 1×10^{13} s^{-1}, 6) W_{U0} was obtained from numerical simulations based on the Scrödinger equation (see below). The broadening for the LPB coupling was taken 18 meV. The photonic SOC for the LPB and UPB were taken from the previous measurements ($T=15$ ps). Exciton and photon lifetimes were taken equal to 5.3 and 15 ps. Parameters used to fit samples 2 and 3 are given in the table in Supplementary Note 2 below.

While the energy relaxation rate W_{ph} may look high, one should remember that the injected particles have to relax down to LPB and decay from the cavity in less than 1 ps, because the Zeeman splitting corresponds to a full pseudospin rotation time of about 2.6 ps, and the observed angles are only a fraction of 2π.

The results of the simulations are shown in Supplementary Figures 1 and 2. Calculations were performed within a detuning range of -20 meV to $+20$ meV, where the mixture of photon and exciton states is always significant. Beyond this detuning range, the polariton states become either very highly excitonic or photonic, which reduces the validity of the model. Detuning values in excess of ±20 meV are therefore not considered. Supplementary Figure 1 shows the linear polarization degree without any applied magnetic field (under linear pumping). Both polariton branches exhibit steady growth of the linear polarization degree when going towards positive detuning. This qualitative dependence for the LPB is explained by the enhancement of the coupling of the reservoir with the LPB: W_L increases with detuning because of the increase of the excitonic fraction, and thus particles spend less time in the reservoir before going to the polariton branches, and therefore have shorter time to lose their polarization degree. Moreover, higher energy of the LPB means better coupling with higher-energy states in the reservoir, where the polarization degree is higher. The UPB, because of the resonant coupling, directly probes the linear polarization degree of the reservoir states. The higher the energy of the state probed, the higher is the linear polarization degree, because the loss of the polarization degree accompanies the energy relaxation of excitons. This is in agreement with the ratio of polarization degrees of LPB and UPB: UPB is stronger polarized, because it is fed from higher-energy states of the reservoir, where the polarization degree is not yet lost.

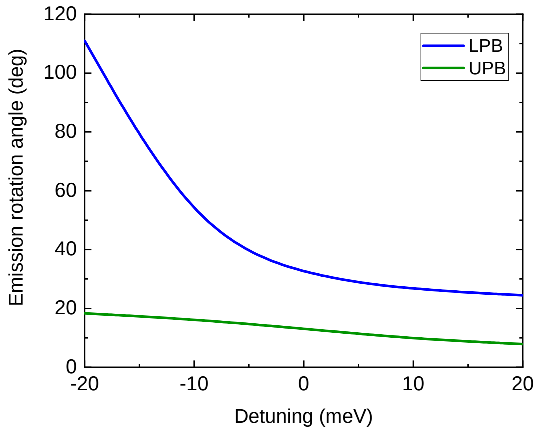

Supplementary Figure 2. Rotation angle of the linear polarization of the polariton modes (blue - LPB, green - UPB), as a function of the exciton-photon detuning.

Supplementary Figure 2 shows the rotation angle of the linear polarization plane under the effect of an applied magnetic field. Both branches show a decrease of the rotation angle with increased detuning, and the angle of the UPB is smaller than that of the LPB. Qualitatively, this behavior is again explained by the time spent by the excitons in the reservoir: the longer is this time, the higher is the rotation angle (simply from the precession equation). For the LPB, positive detuning means better coupling with the reservoir, and so excitons have smaller time to

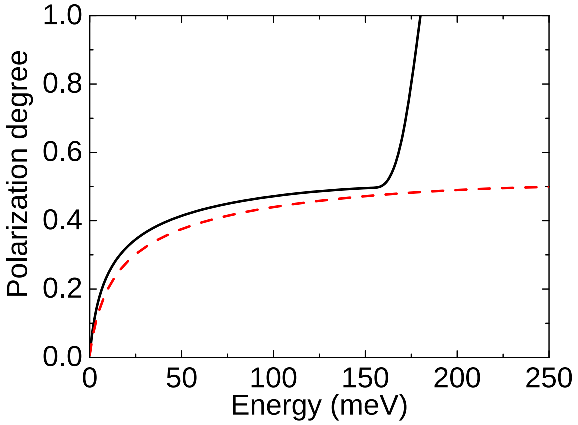

Supplementary Figure 3. Linear polarization degree in the reservoir as a function of energy for 2 different excitation conditions.

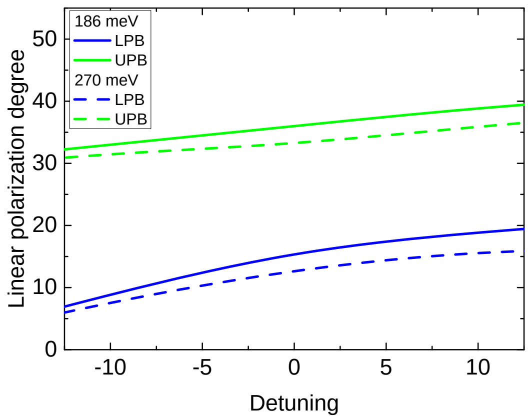

Supplementary Figure 4. Linear polarization degree of the polariton modes: comparison of 2 excitation energies (186 meV - solid line, 270 meV - dashed line).

rotate their linear polarization before they go into the polariton state. The UPB is fed from high-energy states, by the excitons which have rapidly relaxed to these energies and whose polarization plane thus have not yet rotated strongly. Going towards positive detuning means that we probe higher and higher energies, and so the polarization rotation angle becomes smaller and smaller.

Spin relaxation versus energy

The theoretical model of spin relaxation presented above allows to study the dependence of spin relaxation on the energy from different points of view.

While the experiment allows to probe the linear polarization degree in the reservoir via the UPB, resonant with reservoir states, varying the UPB energy requires changing the detuning, which changes the overall relaxation conditions. Theoretical simulations allow to see how the polarization is lost in the reservoir during the energy relaxation for fixed conditions, without changing the detuning. In experiment, this could be checked, for example, by measuring the exciton emission from the side of the sample.

It is also possible to change the energy of non-resonant excitation. In experiments, this has to be chosen at the edge of the cavity stop band, otherwise the injection is not possible. In simulations, we can vary the excitation energy arbitrarily.

Supplementary Figure 3 shows the polarization degree within the reservoir calculated at zero detuning for the same excitation energy as in the experiment (186 meV, black solid line) and for a higher excitation energy (270 meV, red dashed line). In both cases, the polarization is progressively lost during the energy relaxation: the highest values are seen close to the injection energy, while the lowest correspond to the bottom of the exciton reservoir: the longer the particles stay in the system, the more they lose their polarization.

We have also analyzed the dependence of the linear polarization degree emitted from the polariton states at different detunings for pumping at higher energy (270 meV, corresponding to the red curve in Supplementary Figure 3). This could be checked experimentally if it were possible to pump at such frequency, for example, by tuning to another gap of the stop-band. The results are shown in Supplementary Figure 4. We see that pumping at higher energy leads to the decrease of linear polarization degree because of the increase of the energy relaxation time and because the spin-orbit coupling is stronger at higher energies.

Population and polarization lifetimes and broadening of polariton states

The lifetime of the 0D polariton states is determined by several decay rates. First of all, there are the photon lifetime (15 ps) and the exciton non-radiative lifetime (5.3 ps), which gives a polariton lifetime of 7.8 ps at zero detuning for both LPB and UPB. The exciton radiative lifetime is much shorter (of the order of 1 ps), but this emission is supposed to be entirely directed into the cavity mode and taken into account by the Rabi splitting.

Another source of decay for polariton modes (especially the UPB, resonant with high-k excitons) is the scattering of the excitonic fraction on the disorder. This could be naturally estimated using the Fermi's golden rule [1]:

$$\Gamma_{pol\to X} = \frac{2\pi}{\hbar}|V_k|^2\rho(E), \tag{10}$$

where k is the wavevector of a high-k exciton state resonant with the bottom of the polariton dispersion,

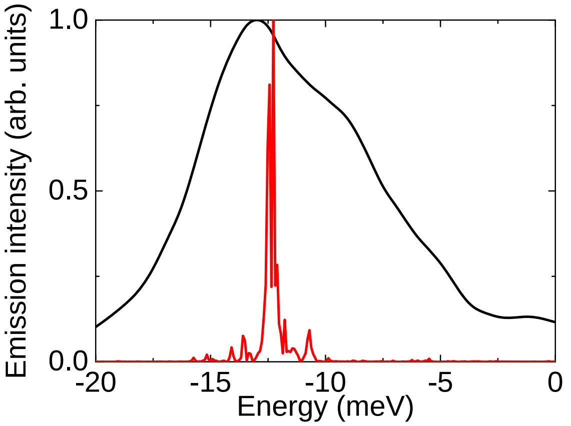

Supplementary Figure 5. Emission spectra of the LPB for two excitation conditions: pulsed optical pumping (black), localized polariton state as initial condition (red).

V_k is the matrix element of the disorder scattering, $|V_k|^2 \propto \exp(-k^2 l_X^2)$ ($l_X = 4$ nm is the disorder correlation length), and $\rho(E)$ is the DOS. We see that the matrix element strongly decreases with the wavevector of the resonant excitonic states k, which provides some protection for the UPB at positive detunings. Indeed, at $\delta = 0$ the UPB is at about 10 meV above the $k = 0$ bare exciton energy, which gives the excitonic wavevector $k = 4.6 \times 10^8$ m^{-1}, leading to a relative decrease of the scattering rate by a factor of 30. Taking into account that only the excitonic fraction is affected by this mechanism, the disorder-limited lifetime can finally be of the order of 7 ps, close to the polariton lifetime estimated above, in spite of the large disorder broadening.

The disorder scattering mechanism contributes to the lifetime if one excites the LPB or the UPB quasi-resonantly, for example, with a relatively long laser pulse. On the contrary, this mechanism does not contribute to the population or coherence decay of the polariton states under non-resonant pumping. Indeed, an exciton from the reservoir, propagating at high k, scatters on the potential, and the final state for this scattering is a localized polariton state (not a plane wave). This localized state already accounts for all disorder present in the system, and therefore the disorder cannot be considered as a perturbation leading to the decay of this state. Therefore, the lifetime of both the UPB and the LPB, and the corresponding coherence time remain unaffected by the possible exchanges between the trapped states and the reservoir.

To describe the case of quasi-resonant excitation quantitatively, we have performed simulations using coupled Schrodinger equations for excitons and photons. The spherical cavity was described by a parabolic potential with corresponding localization scale.

$$i\hbar\frac{\partial\psi}{\partial t} = \hat{T}_{ph}\psi - \frac{i\hbar}{2\tau_{ph}}\psi + U\psi + V\phi + \hat{P} \tag{11}$$
$$i\hbar\frac{\partial\phi}{\partial t} = \hat{T}_X\phi - \frac{i\hbar}{2\tau_X}\phi + U\phi + V\psi$$

Here, ψ is the photon wavefunction, ϕ is the exciton wavefunction, V is light-matter coupling, $\tau_{ph} = 15$ ps is the photon lifetime, $\tau_X = 5.3$ ps is the exciton non-radiative lifetime, U is the disorder potential acting on the excitons with a correlation length $l_X = 4$ nm and rms amplitude $\sigma = 5$ meV. $\hat{P}$ is the pumping operator, corresponding to a Gaussian pumping spot and a quasi-resonant frequency. $\hat{T}_{ph,X}$ are the kinetic energy operators for photons and excitons (with the masses $m_{ph,X}$). These operators are written in the reciprocal space:

$$\hat{T}\psi = F^{-1}\left(\frac{\hbar^2k^2}{2m}F(\psi)\right) \tag{12}$$

where F is the 2D Fourier transform and F^{-1} is the inverse 2D Fourier transform.

We obtain the simulated spectrum of emission from the cavity by a Fourier transform of the wavefunction $\psi(r,t) \rightarrow \psi(k,\omega)$. We plot the spectra for both excitation conditions in Supplementary Figure 5. The black curve is obtained by pulsed optical pumping. It demonstrates a significant broadening due to disorder, which leads to the transfer of particles from the polariton wavepacket towards the excitonic states. The red curve is a spectrum obtained by taking a certain eigenstate as an initial condition at $t = 0$, to simulate an exciton that has been scattered into a localized polariton state. This spectrum is much narrower, indicating that the disorder, already accounted for by the shape of the eigenstate, does not lead to its significant scattering.

SUPPLEMENTARY NOTE 2: COMPARISON OF SAMPLE VARIATION AND FITTING PARAMETERS

Three samples were measured in this investigation, all of which share the same design of a single WSe_2 monolayer transferred onto the surface of a planar dielectric DBR. The results from sample 1 are presented in the main text, and the results from samples 2 and 3 are presented in Supplementary Notes 4 and 5, respectively. The theoretical model presented above was applied to all three samples, and the fitting parameters used for each sample are listed below, along with some relevant experimentally derived properties.

Table of parameters			
Parameter	Sample 1	Sample 2	Sample 3
Valley Zeeman g-factor	-1.7	-4.1	-4.4
Bare exciton PL linewidth	14.5 meV	8.6 meV	9.6 meV
Bare exciton lin. pol. deg.	15 %	23 %	21 %
Bare exciton T_2 time	0.52 ± 0.05 ps	0.48 ± 0.01 ps	Not measured
Rabi splitting	26.2 meV	19.5 meV	21.0 meV
TE-TM spin-orbit coupling β	30 μeV μm	34 μeV μm	34 μeV μm
Energy relaxation W_{ph}	7×10^{14} s^{-1}	10×10^{14} s^{-1}	10×10^{14} s^{-1}
Disorder scattering W_d	2.8×10^{13} s^{-1}	1.8×10^{13} s^{-1}	1.8×10^{13} s^{-1}
Broadening σ	18 meV	12 meV	13 meV
LPB rate W_{L0}	10^{13} s^{-1}	5×10^{13} s^{-1}	5×10^{13} s^{-1}

Several conclusions can be drawn from this list. The main observation which must be stated is that despite sample variation, the retention of valley coherence in polariton states is unambiguously observed in all three samples, with very similar detuning dependences, indicating a universal nature of the cavity-modified exciton relaxation dynamics presented in this work. It is also apparent that the model parameters are very similar across the different samples. Specifically comparing the samples, the first observation is that the Rabi splitting is significantly larger in sample 1 than samples 2 and 3. While sample quality does influence the Rabi splitting, it depends most heavily on the cavity length. A shorter cavity length was used for sample 1 compared to samples 2 and 3, resulting in a larger measured Rabi splitting. Indeed, sample 1 appears to be a more disordered flake compared to the others, as evidenced by the larger PL linewidth and smaller linear polarization degree of the bare exciton. The influence of this disorder means sample 1 has a slower overall energy relaxation, as excitons become trapped in the potential and cannot scatter down so easily. As such, the relaxation rates W_{ph} and W_{L0} are both lower in sample 1 than samples 2 and 3, while the disorder scattering W_d and broadening σ are both larger. It is this slow relaxation which allows sample 1 to have a similar rotation angle of polariton emission under an applied B-field (main text Fig. 5c) as sample 2, despite a smaller valley Zeeman g-factor. We note also that the difference between valley Zeeman g-factors in the three samples is not unusual for WSe_2 samples reported in literature, which vary over a wide range [2, 3], despite an expected value close to -4 [4–6]. The results of the fitting to each sample are shown as overlaid curves on the corresponding experimental data plots in the main text and Supplementary Notes 4 and 5.

SUPPLEMENTARY NOTE 3: ADDITIONAL DATA FROM SAMPLE 1

Additional data from sample 1 (the sample presented in the main text) is displayed in Supplementary Figure 6, including measurement of the valley Zeeman g-factor, and the circular polarization response of both excitons and polaritons. The circular polarization degree of the polariton branches is plotted as a function of exciton photon detuning, $\Delta = E_c - E_{X^0}$, where E_c and E_{X^0} are the cavity and exciton energies respectively, in Supplementary Figure 6**d**. We attribute the high polarization degree of the UPB to direct scattering of highly polarized high k-vector excitons, which have not significantly depolarized, and are degenerate with the UPB energy due to the parabolic exciton dispersion as discussed in detail in [7] for $MoSe_2$ polaritons. The LPB has a polarization degree maximum when the weakly coupled mode is in resonance with the trion at around $\Delta = -30$ meV where the LPB polarization degree is comparable to that of the bare trion state. At positive detuning the LPB approaches the bare exciton polarization degree of around 30%. As discussed in [7] the enhancement of the LPB polarization degree when slightly negatively detuned from the exciton resonance can be explained by taking into account cavity-modified relaxation of excitons, which relax quickly to the polariton branch before significant depolarization in the reservoir due to the exciton LT-splitting.

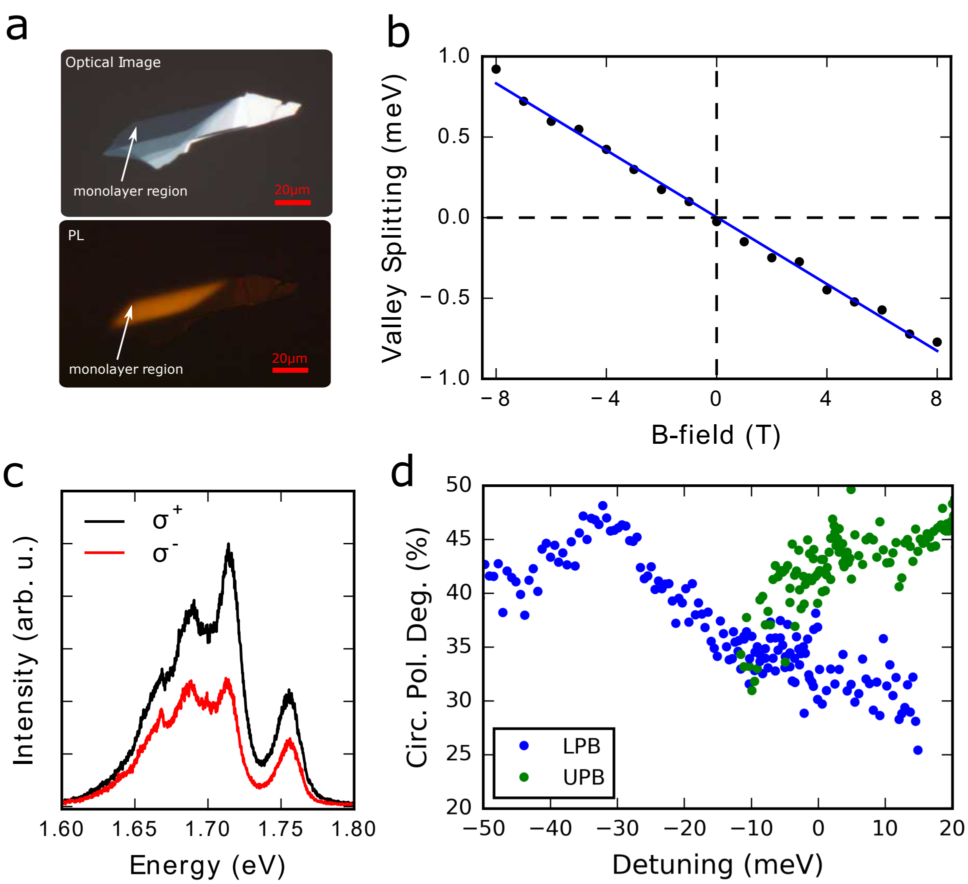

Supplementary Figure 6. **a** (Top panel) microscope image of the monolayer region used in the measurements. (Lower panel) Photoluminescence (PL) map showing the optically active monolayer region of the flake which is around 20x60 μm^2. **b** Valley Zeeman splitting of the neutral exciton as a function of magnetic field, in the scheme $E_{\sigma+} - E_{\sigma-}$. The fit corresponds to a g-factor of -1.7. **c** Circular polarization resolved PL under σ^+ excitation at 1.946 eV. Clear retention of valley polarization is present over the whole spectrum, with circular polarization degrees of 30% and 50% for the exciton and trion respectively. **d** Circular polarization degree of the polariton branches under σ^+ excitation as a function of exciton-photon detuning.

SUPPLEMENTARY NOTE 4: RESULTS FROM SAMPLE 2

All measurements presented in the main text were repeated on a second sample, the results of which are shown below. As can be seen, despite some differences between samples 1 and 2 bare flake properties, such as valley Zeeman g-factor, exciton PL linewidth and polarization rotation angle (Supplementary Figure 7), the main findings from sample 1 are unambiguously reproduced. Strong coupling is achieved between the WSe_2 neutral exciton and cavity mode, with a Rabi splitting of 19.5 meV (Supplementary Figure 8). Valley coherence is observed in exciton-polaritons, with the LPB displaying a linear polarization degree of the order of the bare flake, and the UPB displaying a significantly larger polarization degree (Supplementary Figure 9), in agreement with the model presented in the main text and discussed in detail in Supplementary Note 1. To highlight the detuning dependence of the linear polarization degree of polariton luminescence, polarization resolved spectra are shown at various detunings in Supplementary Figure 10 . Likewise, coherent manipulation of the polariton valley pseudospin vector by applied B-field is reproduced, with UPB and LPB phase rotation angles controllable by exciton-cavity detuning (Supplementary Figure 11).

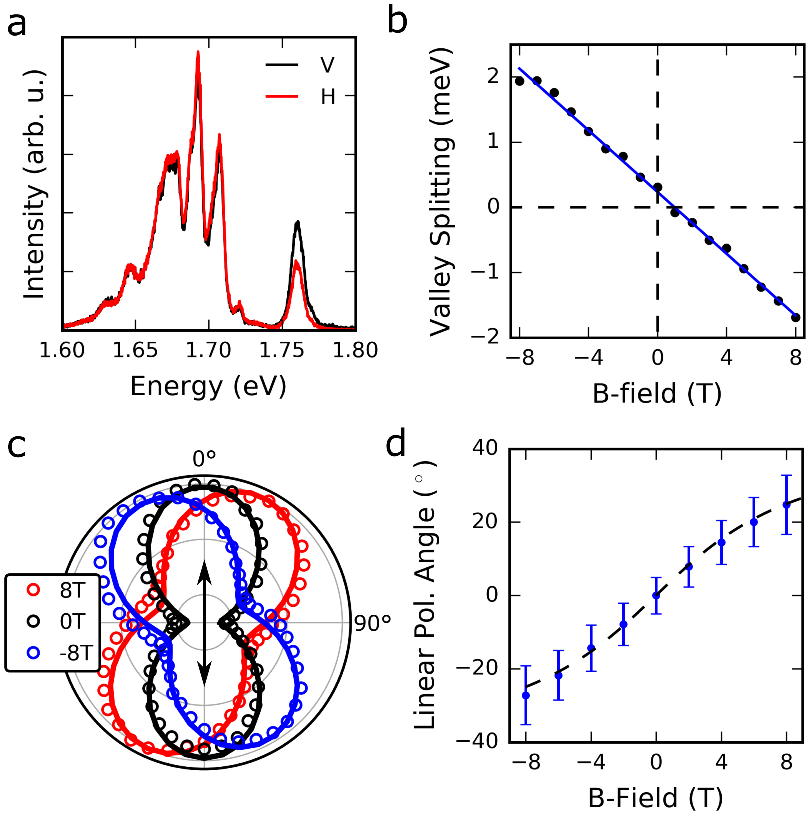

Supplementary Figure 7. **a** Linearly polarized photoluminescence spectra under vertically linearly polarized excitation, showing a bare flake polarization degree of 23%. **b** Valley Zeeman splitting of the neutral exciton. The fit corresponds to a g-factor of -4.1. **c** PL intensity as a function of polarization detection angle from the bare neutral exciton under vertically linearly polarized excitation (denoted by the black arrow) and applied fields of -8 T, 0 T, and +8 T. **d** Linear polarization angle of exciton PL as a function of applied field. The fit corresponds to $T_2 = 0.48 \pm 0.01$ ps, in agreement with sample 1 (see main text for details).

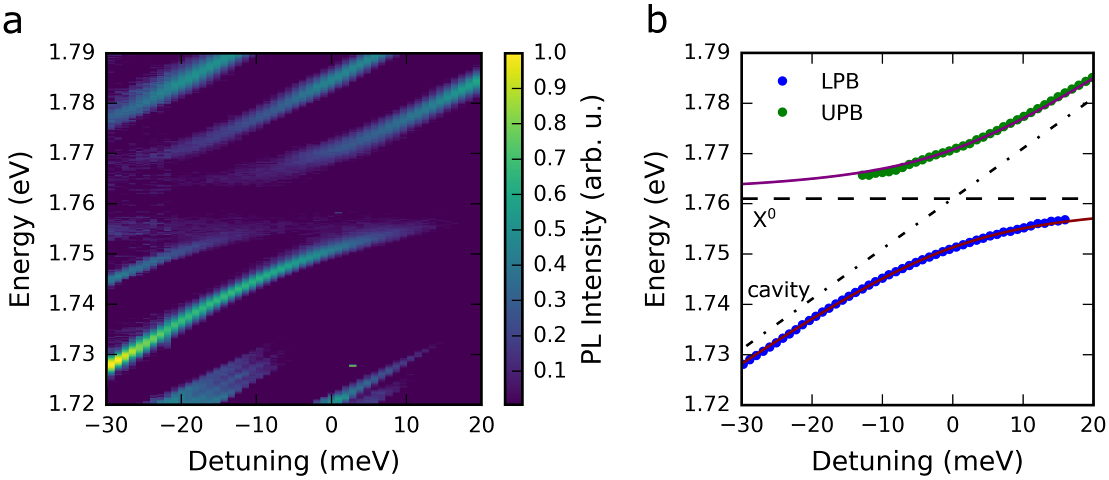

Supplementary Figure 8. **a** Colourmap of polariton photoluminescence as a function of exciton-cavity detuning. A clear anticrossing is visible at the point where the cavity ground state longitudinal mode is in resonance with the neutral exciton, defining spectrally isolated upper and lower polariton branches. Higher order modes are also coupled to the neutral exciton, but these are not considered in this work. **b** Peak positions of the upper and lower polariton branches, extracted by Lorentzian peak fitting, are then fitted to a coupled oscillator model to extract a Rabi splitting of 19.5 meV.

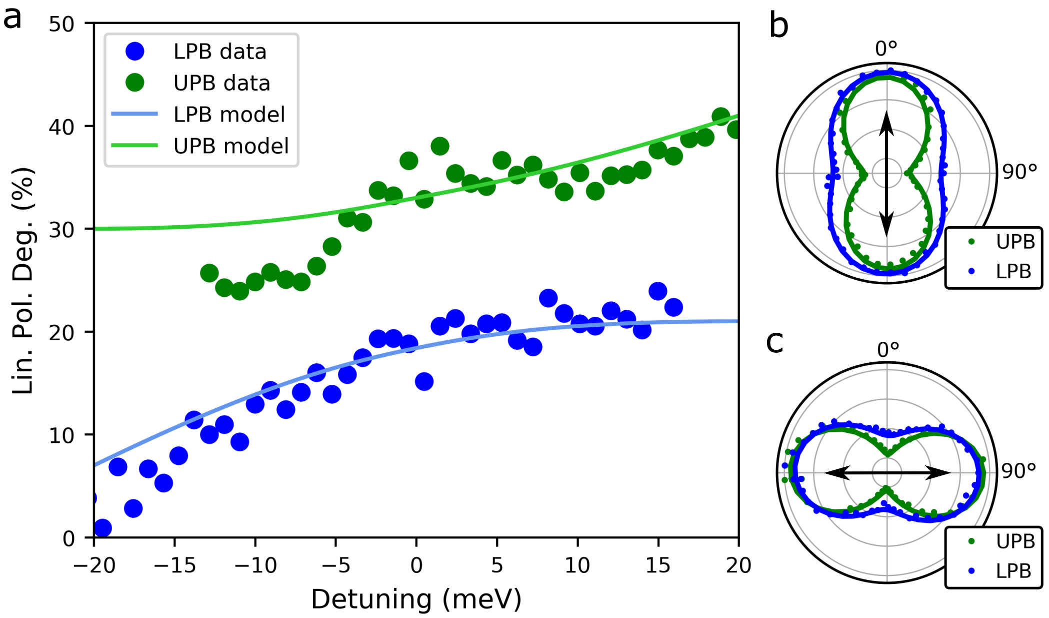

Supplementary Figure 9. **a** Linear polarization degree of the upper and lower polariton branches as a function of detuning. Overlaid curves are simulated using the model discussed in Supplementary Note 1. **b, c** Intensity of polariton PL as a function of detection polarization angle, under (**b**) vertical and (**c**) horizontal excitation polarization. The emission polarization matches the arbitrary angle of the laser, indicating retention of valley coherence.

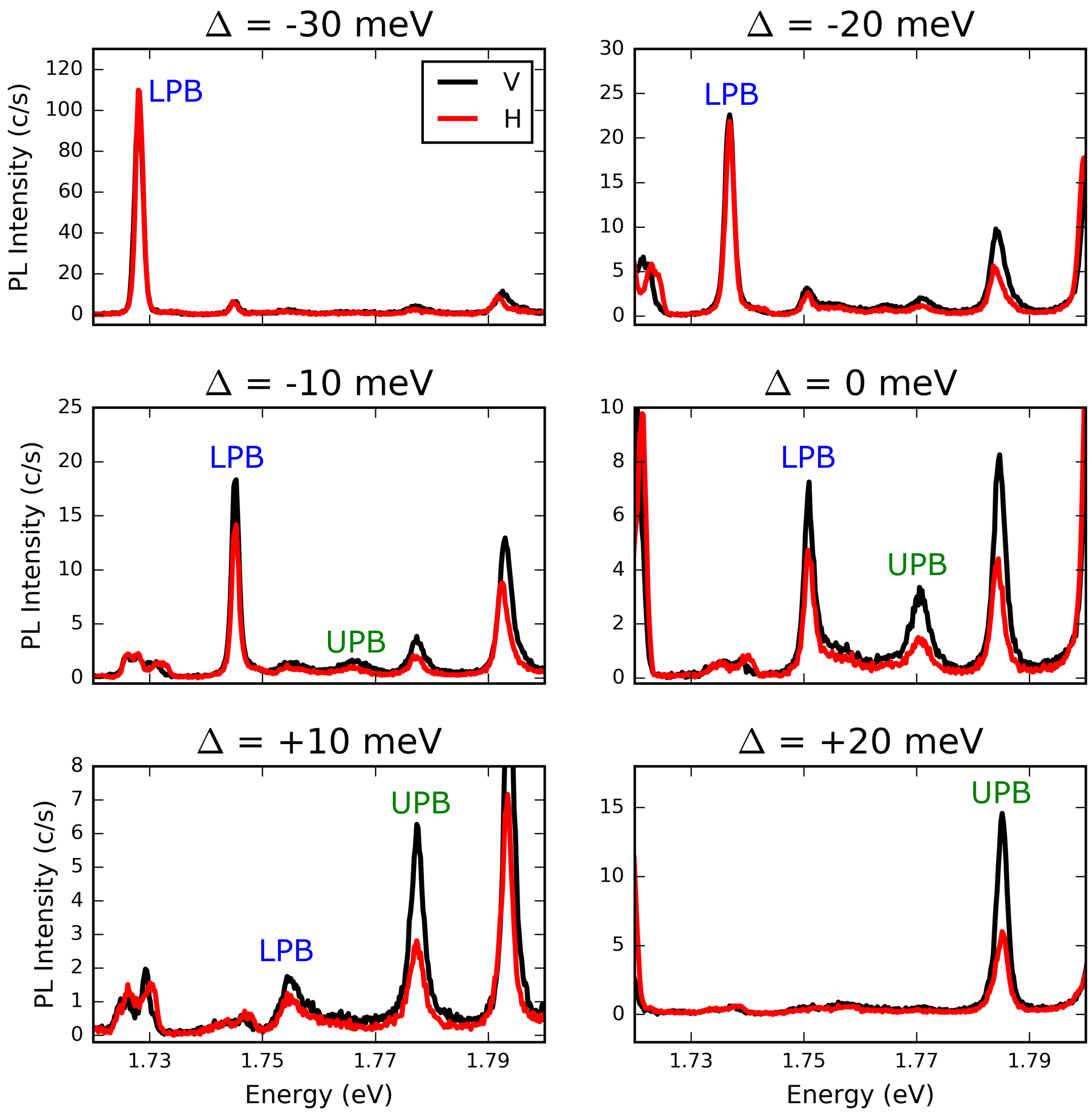

Supplementary Figure 10. Polarization resolved photoluminescence at selected exciton-cavity detunings, under vertically linearly polarized excitation. Clear retention of valley coherence is observed in both the LPB and UPB at exciton-cavity resonance. The lack of polarization in the LPB at strong negative detuning is in stark contrast to the robust valley coherence exhibited by the UPB at positive detuning, indicating the fundamentally different relaxation pathways taken by reservoir excitons into each polariton branch. At positive detunings, the UPB is a robustly valley coherent state with a linewidth much narrower than the bare exciton, and an emission energy tunability of tens of meV.

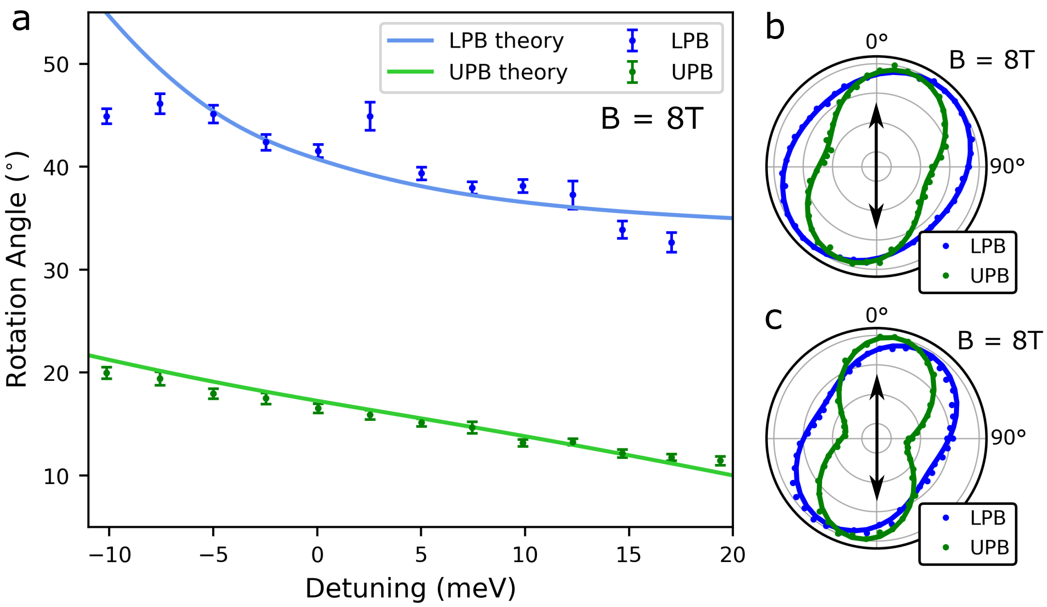

Supplementary Figure 11. **a** Angle of linear polarization of polariton photoluminescence, where 0° corresponds to the vertically polarized excitation, under an external magnetic field of B = 8 T in the Faraday geometry. Rotation of the linear polarization angle corresponds to coherent manipulation of the valley pseudospin vector around the equator of the pseudospin Bloch sphere, as described in the main text. **b, c** PL intensity of both polariton branches as a function of detection polarization angle, under vertically linearly polarized excitation and an applied field of B = 8 T, at detunings of (**b**) −10 meV and (**c**) +17 meV.

SUPPLEMENTARY NOTE 5: RESULTS FROM SAMPLE 3

Linear polarization resolved photoluminescence measurements were carried out in the strong coupling regime with a third sample, the results of which are presented below. The observation of valley coherent exciton-polaritons is reproduced, and the detuning dependence of the linear polarization degree is in agreement with samples 1 (presented in the main text) and 2 (presented in Supplementary Note 4).

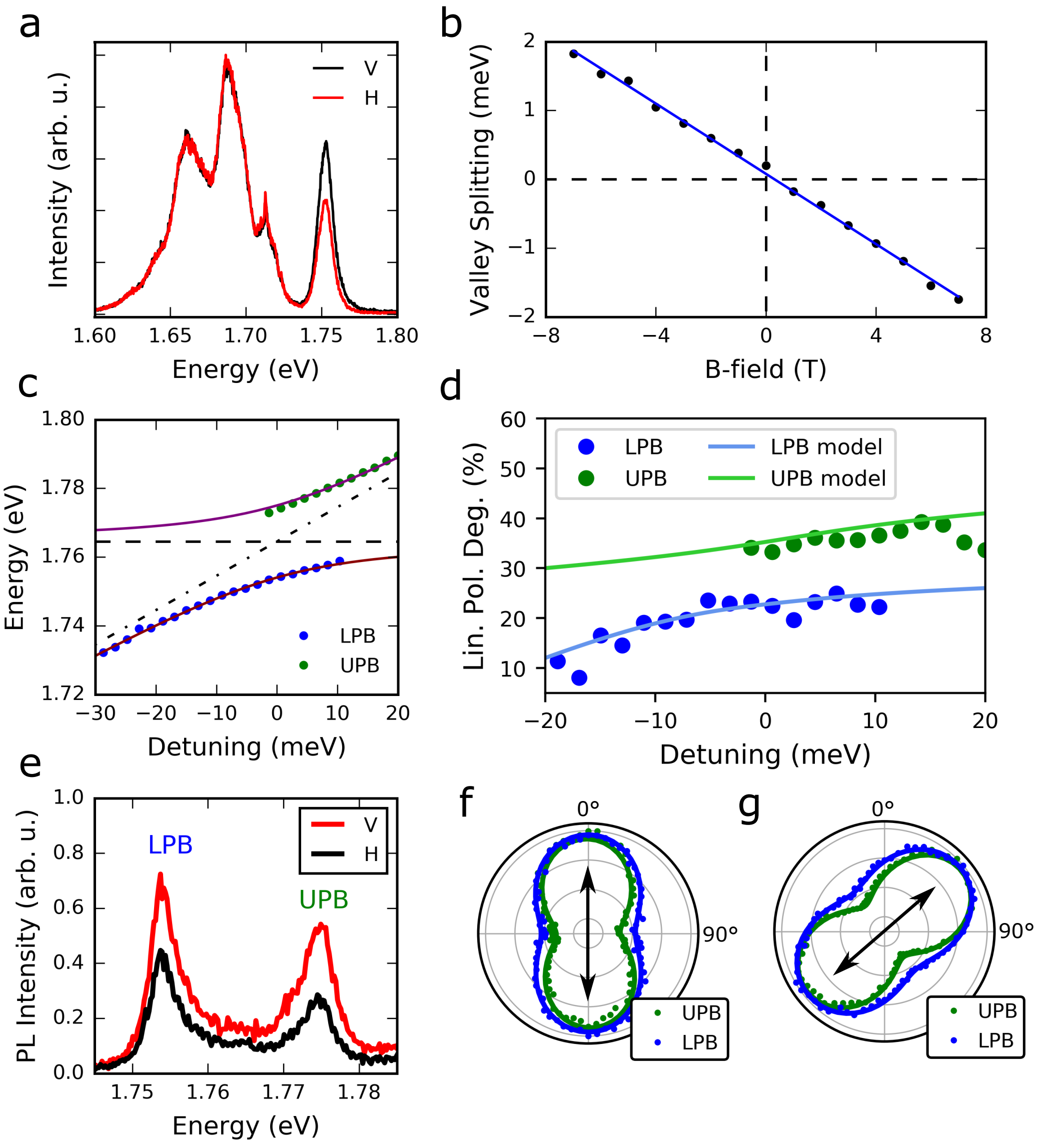

Supplementary Figure 12. **a** Polarization resolved photoluminescence spectra from sample 3 under vertically linearly polarized excitation, showing a bare exciton polarization degree of 21%. **b** Valley Zeeman splitting of the neutral exciton as a function of magnetic field. The fit corresponds to a g-factor of -4.4. **c** Polariton PL from sample 3 embedded in a tunable optical cavity. Peak positions of lower and upper polariton branches are fitted to a coupled oscillator model, giving a Rabi splitting of 21 meV. **d** Linear polarization degree of both polariton branches as a function of exciton-cavity detuning. Clear retention of valley coherence is observed in both the LPB and UPB. The overall trend of the data is in agreement with sample 1 shown in the main text Fig. 3**b**. **e** Polarization resolved polariton spectra under vertically linearly polarized excitation, at zero detuning. Clear retention of optically injected valley coherence is visible in both branches. **f, g** LPB and UPB PL intensity vs detection polarization angle for **(f)** vertically and **(g)** diagonally polarized laser excitation. The polarization orientation of the polaritons matches the laser, indicating initialization of the valley pseudospin vector at arbitrary angles.

SUPPLEMENTARY NOTE 6: LINEAR POLARIZATION ANGLE OF LPB EMISSION

The emission angle of the linearly polarized LPB photoluminescence reflects the intrinsic polarization orientation of the underlying states with which it is quasi-resonant. As such, in the absence of applied fields, at strong negative detuning ~ -40 meV the LPB is quasi-resonant and weakly coupled to the trion, which has a small orthogonal linear polarization (as shown in Fig. 1**b** of the main text), leading to an apparent emission angle approaching 90° (Supplementary Figure 13**c**) when under linearly polarized laser excitation at 0° or 'vertical' polarization. Likewise, when strongly coupled to the neutral exciton at detunings more positive than -10 meV, the LPB emission matches the pump polarization at 0°, corresponding to retention of injected valley coherence. In the detuning range between -30 meV to -15 meV, the LPB lies between trion and exciton energies, and so emits at intermediate polarization angles between 0° and 90°, while displaying a significantly reduced linear polarization degree, as can be seen from the ellipticity in Supplementary Figure 13**d**. As a result of the LPB coupling to the orthogonal trion polarization, any coherent rotation of polariton pseudospin due to the applied B-field is masked at detunings more negative than -10 meV. Consequently, pseudospin rotation measurements are not presented below $\Delta = -10$ meV in Fig. 5**c** of the main text.

The reasons behind the small cross-polarization of the trion remain unclear. It is possible that strong localization effects, whose polarization properties are largely unknown and determined by the local confining potential, are influencing the emission. Alternatively, while it has been suggested that the trion fine structure in WSe_2 causes efficient loss of coherence [8], it has also been reported that the singlet and triplet trion states have different degrees of valley polarization [9], which may lead to a specific response under linearly polarized excitation. A further possibility is the presence of other optical transitions very close to the trion energy, such as phonon replicas and signatures of dark exciton states [10], the polarization properties of which have not been studied in detail.

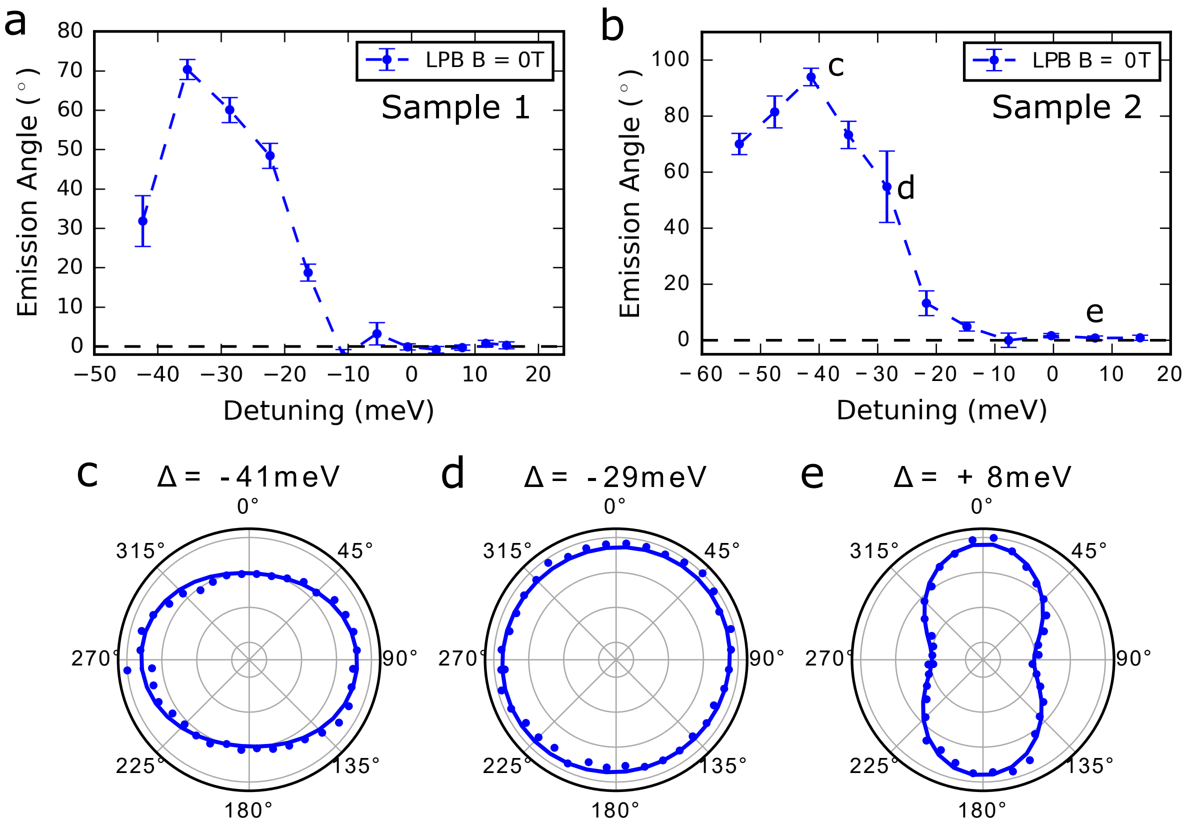

Supplementary Figure 13. **a, b** Emission angle of linearly polarized LPB photoluminescence under vertically (0°) linearly polarized excitation as a function of detuning, at B = 0T, from (**a**) sample 1 and (**b**) sample 2. **c, d, e** Detection angle dependent intensity of LPB PL from sample 2 at (**c**) -41meV (**d**) -29meV and (**e**) +8meV exciton-photon detuning, under vertically linearly polarized excitation. Data points corresponding to each polar plot are labelled on (**b**).

* s.dufferwiel@sheffield.ac.uk
† tplyons1@sheffield.ac.uk
‡ a.tartakovski@sheffield.ac.uk

SUPPLEMENTARY REFERENCES

[1] Bleu, O., Solnyshkov, D. D., Malpuech, G. Optical valley Hall effect based on transition metal dichalcogenide cavity polaritons. *Phys. Rev. B* **96**, 165432 (2017).

[2] Aivazian, G., Gong, Z., Jones, A. M., Chu, R.-L., Yan, J., Mandrus, D. G., Zhang, C., Cobden, D., Yao, W., Xu, X. Magnetic control of valley pseudospin in monolayer WSe_2. *Nat. Phys.* **11**, 148–152 (2015).

[3] Koperski, M., Molas, M. R., Arora, A., Nogajewski, K., Slobodeniuk, A., Faugeras, C., Potemski, M. Optical properties of atomically thin transition metal dichalcogenides: observations and puzzles. *Nanophotonics* **6**, 1289-1308 (2017).

[4] Li, Y., Ludwig, J., Low, T., Chernikov, A., Cui, X., Arefe, G., Kim, Y. D., van der Zande, A. M., Rigosi, A., Hill, H. M., Kim, S. H., Hone, J., Li, Z., Smirnov, D., Heinz, T. F. Valley splitting and polarization by the Zeeman effect in monolayer $MoSe_2$. *Phys. Rev. Lett.* **113**, 266804 (2014).

[5] Stier, A. V., McCreary, K. M., Jonker, B. T., Kono, J., Crooker, S. A. Exciton diamagnetic shifts and valley Zeeman effects in monolayer WS_2 and MoS_2 to 65 Tesla. *Nat. commun.* **7**, 10643 (2016).

[6] Mitioglu, A. A., Plochocka, P., Granados del Aguila, A., Christianen, P. C. M., Deligeorgis, G., Anghel, S., Kulyuk, L., Maude, D. K. Optical investigation of monolayer and bulk tungsten diselenide (WSe_2) in high magnetic fields. *Nano lett.* **15**, 4387-4392 (2015).

[7] Dufferwiel, S., Lyons, T. P., Solnyshkov, D. D., Trichet, A. A. P., Withers, F., Schwarz, S., Malpuech, G., Smith, J. M., Novoselov, K. S., Skolnick, M. S., Krizhanovskii, D. N., Tartakovskii, A. I. Valley-addressable polaritons in atomically thin semiconductors. *Nat. Photon.* **11**, 497–501 (2017).

[8] Jones, A. M., Yu, H., Ghimire, N. J., Wu, S., Aivazian, G., Ross, J. S., Zhao, B., Yan, J., Mandrus, D. G., Xiao, D., Yao, W., Xu, X. Optical generation of excitonic valley coherence in monolayer WSe_2. *Nat. Nanotech.* **8**, 634–638 (2013).

[9] Jones, A. M., Yu, H., Schaibley, J. R., Yan, J., Mandrus, D. G., Taniguchi, T., Watanabe, K., Dery, H., Yao, W., Xu, X. Excitonic luminescence upconversion in a two-dimensional semiconductor. *Nat. Phys.* **12**, 323-327 (2016).

[10] Lindlau, J., Robert, C., Funk, V., Förste, J., Förg, M., Colombier, L., Neumann, A., Courtade, E., Shree, S., Taniguchi, T., Watanabe, K., Glazov, M. M., Marie, X., Urbaszek, B., Högele, A. Identifying optical signatures of momentum-dark excitons in transition metal dichalcogenide monolayers. Preprint: arXiv:1710.00988 (2017).
